# Usage and Exposure to Content of the NHS Healthy Living Program for People With Type 2 Diabetes: Retrospective Observational Cohort Study

**DOI:** 10.2196/89690

**Published:** 2026-06-02

**Authors:** Antonia M Marsden, Rhiannon E Hawkes, Jack S Benton, David P French, Sarah Cotterill

**Affiliations:** 1 Centre for Biostatistics School of Health Sciences University of Manchester Manchester, England United Kingdom; 2 Manchester Centre for Health Psychology School of Health Sciences University of Manchester Manchester, England United Kingdom

**Keywords:** digital interventions, engagement, self-management, type 2 diabetes, usage data

## Abstract

**Background:**

Diabetes self-management education and support (DSMES) programs can improve health outcomes, but engagement is often low. “Healthy Living” is a web-based self-management program for people with type 2 diabetes, based on the “HeLP-Diabetes” intervention, which demonstrated effectiveness in a randomized controlled trial. Healthy Living was commissioned by the National Health Service in England and rolled out nationally into routine care in 2020. The program comprises web-based structured learning, unstructured articles (which users could access at any time), and tracking tools such as goal setting. It is important to assess not only the uptake of digital interventions but also the amount of time spent using the intervention and the content they engage with. There is currently limited research on the extent to which people engage with digital DSMES program content outside of a trial setting.

**Objective:**

This study aimed to investigate the overall usage and exposure to content of Healthy Living, including differences in usage/exposure by user characteristics.

**Methods:**

This study used a retrospective observational cohort study design to conduct a service evaluation of Healthy Living. Pseudonymous usage data from all people with type 2 diabetes (N=27,422) who activated a Healthy Living account between May 2020 and September 2023 were available, including (1) which program activities were accessed, (2) when activities were accessed, and (3) how long users spent on each activity. User demographic and usage information was summarized using means and SDs, medians and IQRs, or frequencies and percentages. Logistic regression evaluated the association between user demographics and usage.

**Results:**

Of the 27,422 users who activated a Healthy Living account, the mean age was 58.6 (SD 12.0) years, and the percentage of females was 56.8% (15,385/27,422). There was an even spread across deprivation quintiles. The median length of time spent on the program in total was 7.6 (IQR 0.6-27.6) minutes; 12,066 (44%) users spent <5 minutes on the program, and 3022 (11%) spent ≥1 hour. Of those who activated an account, 69.8% (19,137/27,422) accessed some program content, 40.7% (11,149/27,422) completed the first section of structured education, and 4.7% (1219/27,422) completed 60% of the structured education. Usage of the unstructured aspects of the program was low. Female gender, lower deprivation, White ethnicity, and a shorter time since diagnosis were associated with increased usage.

**Conclusions:**

This study is one of the first to provide detailed analysis of user engagement with a national digital self-management program for type 2 diabetes. Unlike previous evaluations conducted on smaller sample sizes, this study highlights the low usage of Healthy Living across >27,000 users. Given that higher usage of DSMES programs is associated with improved health outcomes in people with type 2 diabetes, further work needs to identify how to encourage increased engagement with the program.

## Introduction

### Overview

Type 2 diabetes is a chronic condition that requires long-term management [1]. Diabetes self-management education and support (DSMES) aims to inform people about their condition, for example, through providing guidance on diet, physical activity, blood glucose control, and medication, to enable people to manage their diabetes on an ongoing basis, thus reducing their risk of further health and physiological problems [2,3]. In the United Kingdom, the National Institute for Health and Care Excellence (NICE) guidelines outline that DSMES should be offered to all adults newly diagnosed with type 2 diabetes [1]. There is evidence that such programs can result in reduced hemoglobin A_1c_ (HbA_1c_; blood glucose) levels, reduce the risk of complications, increase quality of life, and foster positive behavioral changes relating to exercise and nutrition [4,5].

Despite the potential benefits, attendance for DSMES can be low. Data from the 2022 National Diabetes Audit in the United Kingdom estimates that approximately 76.3% of people newly diagnosed with type 2 diabetes were offered structured education within 12 months of their diagnosis, but only 8.6% attended within the first 12 months [6]. Such interventions are typically delivered as face-to-face group-based courses [7,8]. However, digital interventions, delivered via websites or apps, are potentially more appealing to some, including (1) those who work and/or have caring responsibilities [9], (2) those with severe mental illness or intellectual disability, and (3) ethnic minority groups [10]. To widen access to support for people with type 2 diabetes in England, the National Health Service (NHS) 10-Year Plan has committed to shifting care from analog to digital, by expanding access to digital self-management tools and DSMES programs [11]. All demographic groups should be involved in developing digital services, and tailoring can be helpful to minimize digital inequality [10]. Systematic review evidence suggests that digital DSMES can be effective in improving HbA_1c_ in patients with type 2 diabetes [12].

HeLP-Diabetes was an online DSMES designed to provide information about type 2 diabetes and its treatments, and support people to make and retain lifestyle changes in areas such as physical activity and diet [13]. A randomized controlled trial (RCT) comparing the HeLP-Diabetes intervention to a simple information website demonstrated modest but statistically significant reductions in HbA_1c_ at 12 months for those allocated to the intervention (mean difference between groups at 12 months: –2.6, 95% CI –4.8 to –0.5 mmol/mol; *P*=.01) [14]. HeLP-Diabetes was shown to be cost-effective [15].

A national rollout of HeLP-Diabetes in routine care was commissioned by the NHS in England in 2019 [16]. The program, named “Healthy living for people with type 2 diabetes,” or “Healthy Living” for short, was modified and delivered by an external provider for the NHS, but showed high fidelity of content to the version evaluated in the trial [17]. The program underwent beta testing between May 2020 and June 2021, followed by the full-scale rollout. Healthy Living is one of several DSMES programs available in the United Kingdom, alongside structured group options such as DESMOND and X-PERT, which offer face‑to‑face group education and self‑management support delivered by trained educators in local communities, often complemented by a digital app. People with type 2 diabetes can be referred to any of these services to help them manage their condition, although access depends on what is commissioned locally. Healthy Living was designed not to replace existing provision, but to provide a universally available, free, evidence‑based online alternative that expands patient choice beyond what is offered in their local area.

It is important to assess not only the uptake of digital interventions, but also the engagement once participants have enrolled, for example, the amount of time spent using the intervention and the content they engage with [18]. This is to (1) examine why there may be differences in outcomes in routine delivery compared to an RCT context, (2) inform whether there is a need to improve engagement of particular elements of the intervention, and (3) examine whether less usage in disadvantaged groups exacerbates inequalities. For example, if users do not engage with the intervention, they will not be exposed to important intervention content to help with self-management of their condition, which could result in reduced health outcomes. Further, while there is evidence that low socioeconomic status groups are significantly less likely to start using chronic disease self-management support interventions, the literature is unclear on whether low socioeconomic status is related to retention and usage, with only a few small studies [19]. Analysis of program usage is needed, including understanding how it varies among the population, so programs can be tailored to underrepresented groups [10] and avoid exacerbating health inequalities.

Previous studies investigating engagement with DSMES programs for type 2 diabetes have shown mixed results. In the HeLP-Diabetes RCT, the mean number of log-ins for users of the intervention was 18.7 (SD 84.0), and the mean number of days in which the website was accessed over the 12-month follow-up period was 10.1 (SD 22.9) [14]. In an RCT of the BetaMe online program in New Zealand, initial engagement was high, with 74% of participants having active engagement within the first 16 weeks, but this declined over time [20]. However, levels of usage in RCT settings may be an overestimation of that in real-world settings; a systematic review found that the real-world usage rate of unguided e-mental health programs was, on average, 4 times lower than that reported in the RCTs of the same programs [21]. There has been less research on engagement with DSMES based on real-world data, but one study is the observational analysis of usage of a UK-based DSMES program for type 2 diabetes, myDESMOND, where 5360 (56.3%) of 9522 users remained on the program for at least 1 month, and 1676 (17.6%) remained on for 1 year [22].

### Objectives

This study was able to investigate engagement with a nationally implemented DSMES program in a sample of routine users of Healthy Living. The aim of this work was to summarize usage of the Healthy Living program, specifically investigating (1) overall usage (such as how often participants accessed the self-management program and how long they spent on the program), (2) what program content was accessed by participants, and (3) how usage varied across demographics.

## Methods

### Design

This study was part of a wider service evaluation of Healthy Living, in which all data from users of the Healthy Living program were analyzed. Thus, a retrospective observational cohort design was used, using pseudonymized participant-level usage data collected by the Healthy Living service provider linked to the National Diabetes Audit [6].

### Healthy Living Program

The Healthy Living program is a web-based self-management program, accessible on computers, smartphones, and tablets, with 3 components: the “Learn Journey,” “Find Answers,” and “Tools.” The Learn Journey, the main component, contained structured information comprising 1-page articles containing a mixture of text, pictures, videos, and quizzes, grouped into sections. At the time of this evaluation, there were >30 sections of the Learn Journey which users were required to work through in a defined order. The majority of the Learn Journey was the same for all users, but there was additional information on smoking, driving, and working for those who indicated this was relevant. NHS England defines “attendance” on the program as reaching the end of the first section in the Learn Journey (“Introduction to type 2 diabetes”), and “completion” of the program as accessing 60% of the Learn Journey (see Textbox 1 for definitions).

The Find Answers component was unstructured and comprised articles, grouped in sections, that users can access at any time. The “Tools” section comprised 5 tracking tools users could access at any time: goal setting, blood test tracking, food tracking, weight tracking, and step tracking. See Multimedia Appendix 1 for screenshots of the 3 main components of the Healthy Living intervention at the time of the evaluation.

**Definitions of Healthy Living program components.**

**Learn Journey**

- Structured information comprising 1-page articles containing a mixture of text, pictures, videos, and quizzes, grouped into sections.

**Find Answers**

- Unstructured information comprising articles, grouped into sections, that users could access at any time.

**Tools**

- Section comprises 5 tracking tools users could access at any time: goal setting, blood test tracking, food tracking, weight tracking, and step tracking.

**Session**

- A string of continuous activity on the program, ending when the user was inactive for over 10 minutes.

**Attendance**

- Reaching the end of the first section in the Learn Journey.

**Completion**

- Accessing at least 60% of the Learn Journey.

### Participants

Participants were referred to Healthy Living through their general practice or via self-referral. To register, users visited the Healthy Living website and provided their name, type 2 diabetes status (ie, whether they were a patient, caregiver, or health care professional), email address, gender, date of birth, postcode, and how they heard about Healthy Living. Following registration, users were sent an email with a link to complete their profile and activate their account, after which they could access program content.

### Setting and Study Size

This study includes data from all users who activated an account between May 11, 2020, and September 1, 2023, and had a type 2 diabetes diagnosis. In cases where individuals had multiple active accounts, we selected the account from which the user had accessed the highest number of educational articles, and if the number was equal, we selected the latest account. Users who activated an account less than 1 month before the date of data extraction (September 1, 2023) were excluded, as they may not have had sufficient time to work through the materials. The suitability of this 1-month time period was investigated in a sensitivity analysis.

### Variables and Data Measurement

#### Personal Characteristics

In addition to the user information provided by participants at registration, information on ethnicity and time since diagnosis of type 2 diabetes was obtained from the National Diabetes Audit [6]; users were linked using a pseudonymized NHS number. However, this information was unavailable for those with a missing pseudonymized NHS number (2237/27,422, 8.2% of users). Other information, such as first language, disability status, and smoking status, was collected from users once they had activated their account, but this has not been included due to high levels of missingness (>50%). Postcode entered at registration was used to determine the 2019 Index of Multiple Deprivation quintile [23].

#### Program Usage

Data on activities undertaken on the Healthy Living program by all users was collected: when users logged in, what articles in the Learn Journey and Find Answers sections were accessed, what tools were accessed, when the articles and tools were accessed, and the amount of time spent on each activity. This enabled us to summarize across all users.

#### Summaries of Overall Usage

The following measures were used to summarize overall program usage:

1. The amount of time spent on the program at each session and the total amount of time spent on the program.
2. The number of program “sessions” attended, where a session is defined as a string of continuous activity on the program, ending when the user was inactive for over 10 minutes.
3. The number of users who accessed program content.
4. The number of users who “attended” the program, where attendance is defined as reaching the end of the first section in the Learn Journey.
5. The number of users who “completed” the program, where completion is defined as accessing at least 60% of the Learn Journey (ie, reaching the end of section 15).
6. When sessions took place.

#### Summaries of Program Content Accessed

The following measures were used to summarize access to program content:

1. The number of sections in the Learn Journey accessed.
2. The number of articles in the Find Answers section accessed.
3. The most accessed articles in the Find Answers section.
4. How often the tools were used.

#### Bias

Note that for the amount of time spent on the program at each session and the total amount of time spent on the program, because of the way in which a session was defined, the duration of the last activity of each session was unknown. These missing durations were set to the median duration of activities of the same type (eg, Learn Journey article, Find Answers article, and step tracker access) undertaken by the user, if available. If this median could not be calculated because the user had no other observations of the same type, the missing duration was set to the median duration of activities of the same type undertaken by all other users.

### Statistical Methods

User characteristics and usage information were summarized using means and SDs, medians and IQRs, or frequencies and percentages, as appropriate. User characteristics were also summarized separately in those who did and did not “attend” the program and those who did and did not “complete” the program. Logistic regression was used to estimate the association between each usage characteristic with both “attendance” and “completion,” while adjusting for the other characteristics. Analyses were performed in Stata (version 17; Stata Corp LLC).

### Ethical Considerations

This study was reviewed and approved by the Yorkshire and the Humber-Leeds West NHS Research Ethics Committee (reference number 20/YH/0250). All users of Healthy Living had provided consent for their pseudonymized data to be accessed for service evaluations, and agreed to the terms and conditions of the privacy policy before they accessed the program. Data presented in this manuscript are pseudonymized. As this was a service evaluation, users were not compensated as they did not actively participate in any research, and instead, only their pseudonymized data were obtained. No identification of individual participants/users in this manuscript or supplementary material is possible.

## Results

### Participant Characteristics

**Figure 1:**
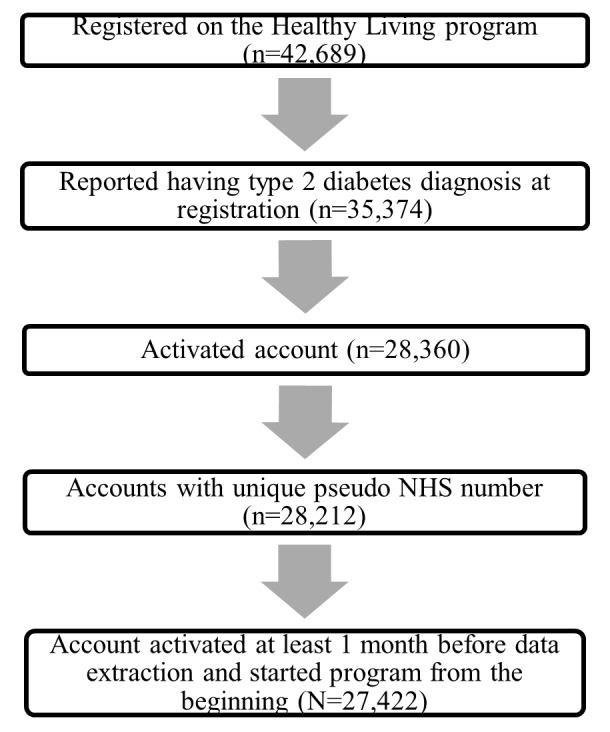
Flow diagram of participant selection for inclusion in analysis.

Figure 1 describes participant selection. Data from 42,689 users who registered on the program were obtained, and 27,422 of these were determined to have reported a type 2 diabetes diagnosis at registration and activated a Healthy Living account more than 1 month before data extraction, that is, before September 1, 2023. The sensitivity analysis suggested that increasing this period to 3, 6, and 9 months made little difference to the findings (Multimedia Appendix 2). Note that, while the pseudonymized NHS number was used to identify accounts belonging to the same person, these data were missing for 8.2% (2237/27,422) of eligible participants who activated an account, so some duplicates may have been missed.

Table 1 summarizes characteristics of the 27,422 users. The mean age was 58.6 (SD 12.0) years, and the percentage of females was 56.8% (15,385/27,088). There was an even spread across deprivation quintiles. The majority of users were made aware of Healthy Living through a health care professional from their general practitioner practice (12,178/27,422, 44.4%), social media (5053/27,422, 18.4%), or an online search (4758/27,422, 17.4%). Users self-referred in nearly all cases, 15.6% (4282/27,422) doing so through a private link before the program was publicly available, and 81.9% (22,467/27,422) doing so once the program was public. Regarding ethnicity, 84.9% (17,628/20,755) of those with a nonmissing entry were White, 8.8% (1835/20,755) were Asian, 4.2% (862/20,755) were Black, 1.1% (228/20,755) were mixed, and 1% (202/20,755) were classed as “other.” The median number of years since diagnosis was 3 (IQR 0-10) years.

**Table 1:** Descriptive characteristics of users with type 2 diabetes who activated a Healthy Living account between May 2020 and September 2023 (N=27,422).

Characteristics		Values
Age (years)		
	Mean (SD)	58.6 (12.0)
	Median (IQR)	59 (51-67)
	Missing, n (%)	139 (0.5)
Gender, n (%)		
	Male	11,644 (43)
	Female	15,385 (56.8)
	Intersex	59 (0.2)
	Missing	334 (1.2)
IMD^a^ quintile, n (%)		
	1 (most deprived)	4920 (18.9)
	2	5029 (19.3)
	3	5403 (20.7)
	4	5601 (21.5)
	5 (least deprived)	5129 (19.7)
	Missing	1340 (4.9)
Ethnicity, n (%)		
	Asian	1835 (8.8)
	Black	862 (4.2)
	Mixed	228 (1.1)
	Other	202 (1)
	White	17,628 (84.9)
	Missing	6667 (24.3)
Years since diagnosis		
	Mean (SD)	6.3 (6.9)
	Median (IQR)	4 (0-10)
	Missing, n (%)	5961 (21.7)
How the user was made aware of Healthy Living, n (%)		
	Charity or community group-Diabetes UK	700 (2.6)
	Charity or community group-other	76 (0.3)
	Friend or family member	504 (1.8)
	Health care professional-GP^b^ practice	12,178 (44.4)
	Health care professional-other	680 (2.5)
	Online search	4758 (17.4)
	Poster, leaflet, or other NHS^c^ promotion	1381 (5)
	Social media	5053 (18.4)
	None of the above	2092 (7.6)
	Missing	0
Referral route, n (%)		
	Public beta-Hub referral (NWL^d^)^e^	323 (1.2)
	Self-referral landing page (private)	4282 (15.6)
	Self-referral landing page (public)	22,467 (81.9)
	GP referral	350 (1.3)
	Missing	0

^a^IMD: Index of Multiple Deprivation. IMD scores associated with the lower super output area derived from venue postcodes, ranging from the most deprived areas in England to the least deprived areas in England.

^b^GP: general practitioner.

^c^NHS: National Health Service.

^d^NWL: North West London.

^e^The option “Public beta-Hub referral (NWL)” refers to referrals via an online learning platform targeted at primary care staff in North West London.

### Summary of Overall Usage

Table 2 summarizes overall usage of the program. The median length of time spent on the program by a total of 27,422 users who activated their accounts was 7.6 (IQR 0.6-27.6) minutes; 12,066 (44%) of users spent less than 5 minutes on the program, and 3022 (11%) spent 1 hour or more on the program. Over half completed only 1 session (15,089/27,422, 55%), and 3171 (11.6%) completed 5 or more sessions. Approximately 70% (n=19,137) accessed some program content at least once, 40.7% (n=11,149) “attended” the program, and 4.7% (n=1291) “completed” the program (see Textbox 1 for definitions).

**Table 2:** Summary of overall usage of the Healthy Living program between May 2020 and September 2023 (N=27,422).

			Values
Total time spent on the program in minutes			
	<1 minute, n (%)	7598 (27.7)	
	1-5 minutes, n (%)	4468 (16.3)	
	5-30 minutes, n (%)	8971 (32.7)	
	30-60 minutes, n (%)	3363 (12.3)	
	60-120 minutes, n (%)	2025 (7.4)	
	>120 minutes, n (%)	997 (3.6)	
	Median (IQR)	7.6 (0.6-27.6)	
	Mean (SD)^a^	24.5 (58.4)	
Total number of sessions^b^			
	1, n (%)	15,089 (55)	
	2-4, n (%)	9162 (33.4)	
	5-9, n (%)	2183 (8)	
	>10, n (%)	988 (3.6)	
	Median (IQR)	1 (1-3)	
	Mean (SD)^a^	3.1 (19.9)	
Accessed some program content (Learn Journey, Find Answers, or Tools), n (%)			19,137 (69.8)
“Attended” the program^c^, n (%)			11,149 (40.7)
“Completed” the program^d^, n (%)			1219 (4.7)

^a^The mean is presented to aid comparison with similar programs. The median is our preferred summary measure due to the skewed distribution of these variables.

^b^A session is defined as a string of continuous activity on the program, ending when the user was inactive for over 10 minutes.

^c^Attendance is defined as reaching the end of the first section in the Learn Journey.

^d^Completion is defined as accessing at least 60% of the Learn Journey.

In total, 83,731 sessions were recorded over 60,830 person-days. Table 3 summarizes the time of day and day of week that sessions took place. The most popular time of day for sessions was 12 PM-6 PM (3887/83,731, 35.3% of sessions), although the number of sessions starting between 6 AM-12 PM and 6 PM-12 AM was only slightly lower (approximately 30% in each). Weekends were slightly less popular than weekdays. Of the 83,731 sessions, 32,032 (38.3%) started between 9 AM and 5 PM on Monday-Friday.

**Table 3:** Summary of time of day and day of week that users completed sessions^a^ on the Healthy Living program between May 2020 and September 2023.

			Values, n (%)		
Time of day (n=83,731)					
	12 AM-6 AM	3887 (4.6)			
	6 AM-12 PM	27,017 (32.3)			
	12 PM-6 PM	29,539 (35.3)			
	6 PM-12 AM	23,288 (27.8)			
Day of the week (n=60,830)					
	Monday	10,017 (16.5)			
	Tuesday	9805 (16.1)			
	Wednesday	9625 (15.8)			
	Thursday	9560 (15.7)			
	Friday	9300 (15.3)			
	Saturday	6301 (10.4)			
	Sunday	6222 (10.2)			

^a^A session is defined as a string of continuous activity on the program, ending when the user is inactive for over 10 minutes.

### Summary of Program Content Accessed

Table 4 summarizes the number of sections in the Learn Journey accessed and the number of articles in the Find Answers section accessed by the 19,137 users who accessed program content.

The median number of Learn Journey sections accessed was 2 (IQR 1-4). A total of 10,273 (53.7%) users accessed 1-3 sections, and 1090 (5.1%) accessed 20 or more sections. Table S4 in Multimedia Appendix 3 lists each section of the Learn Journey and the number of people who accessed it. Seven articles in the Learn Journey had a median duration of engagement of 90 seconds or more; 6 of these included interactive quizzes, and 1 contained an embedded video. These are listed in Multimedia Appendix 4.

Of the 19,137 users, 5825 (30.4%) accessed the Find Answers section at least once, and the median number of articles accessed was 0 (IQR 0-1); 1453/19,137 (7.6%) accessed only 1 article in total, and 1814/19,137 (9.5%) accessed over 10 articles. The most accessed articles were in the “Eating well for diabetes” section; this is the second section that appears in this part of the program, after the section “About type 2 diabetes.” The 5 most popular articles were: “Meals and portion sizes” (accessed by n=1148, 6%), “Foods to eat and avoid” (n=1091, 5.7%), “Healthy portion plate” (n=844, 4.4%), “Fruit and vegetables” (n=839, 4.4%), and “Lower carbohydrate plate” (n=832, 4.4%). All articles that were accessed by more than 600 users are listed in Multimedia Appendix 5 (Table S5).

Table 5 shows how many users accessed each of the tools in the Tools section. Of the 19,137 users, 3066 (16%) visited at least one of the tool sections once or more, but there was little evidence of regular use of the tracking tools.

**Table 4:** Summary of the number of sections in the Learn Journey and the number of articles in the Find Answers section that were accessed by Healthy Living users between May 2020 and September 2023 (n=19,137).

		Values
Number of sections of the Learn Journey accessed		
	0 sections, n (%)	1483 (7.8)
	1 section, n (%)	6829 (35.7)
	2-3 sections, n (%)	3444 (18)
	4-5 sections, n (%)	4246 (22.2)
	6-9 sections, n (%)	1233 (6.4)
	10-19 sections, n (%)	812 (4.2)
	20-29 sections, n (%)	1090 (5.7)
	Median (IQR)	2 (1-4)
	Mean (SD)^a^	4.1 (5.7)
Number of articles in the Find Answers section accessed		
	0 article, n (%)	13,312 (69.6)
	1 article, n (%)	1453 (7.59)
	2-4 articles, n (%)	1577 (8.2)
	5-9 articles, n (%)	981 (5.1)
	10-19 articles, n (%)	847 (4.4)
	20+ articles, n (%)	967 (5.1)
	Median (IQR)	0 (0-1)
	Mean (SD)^a^	2.9 (8.8)

^a^The mean is presented to aid comparison with similar programs. The median is our preferred summary measure due to the skewed distribution of these variables.

**Table 5:** Summary of the number of Healthy Living users who accessed each of the tools on at least 1, 2, and 3 separate days between May 2020 and September 2023.

	Accessed on at least 1 day (n=19,137), n (%)	Accessed on at least 2 days (n=19,137), n (%)	Accessed on at least 3 days (n=19,137), n (%)
Goals	2303 (12)	326 (1.7)	53 (0.3)
Weight tracking	1612 (8.4)	520 (2.7)	312 (1.6)
Food tracking	248 (1.3)	61 (0.3)	34 (0.2)
Step tracking	211 (1.1)	100 (0.5)	71 (0.4)
Blood test tracking	490 (2.6)	151 (0.8)	104 (0.5)
Any tool	3066 (16)	787 (4.1)	425 (2.2)

### Usage by User Characteristics

Table 6 summarizes user characteristics separately by “attendance” and “completion” of the program, and gives the results of the logistic regression analyses.

“Attendance” was associated with ethnicity: those with an Asian, Black, or mixed ethnicity had lower odds of “attending” the program compared to those with a White ethnicity. Additionally, male gender, being from a more deprived area, a longer time since diagnosis of type 2 diabetes, and being made aware of Healthy Living via social media, compared with being made aware via a health care professional, were all associated with reduced odds of “attendance.” Although statistically significant, the odds ratio for age regarding “attendance” was 1.00 (95% CI 1.00-1.001), suggesting a minimal association.

Associations with “completion” were similar to those for attendance, although the CIs were wider due to a relatively small number of participants “completing” the program. Male gender, being from a more deprived area, Asian, Black, or mixed ethnicity (compared to White ethnicity), a longer time since diagnosis of type 2 diabetes, and being made aware of Healthy Living via social media (compared to being made aware via a health care professional), were all associated with reduced odds of “completion.”

**Table 6:** Attendance and completion of Healthy Living between May 2020 and September 2023 summarized by user characteristics with odds ratios from logistic regression analyses.

			Attendance (n=11,149)^a^											Completion (n=1291)^b^							
			Summary, n (%)					Logistic regression					Summary, n (%)						Logistic regression		
			“Attended” program		Did not “attend” program		Odds ratio (95% CI)			*P* value		“Completed” program				Did not “complete” program		Odds ratio (95% CI)			*P* value
Gender																					
	Male (baseline)	4220 (38.5)		7424 (46.2)		N/A^c^			N/A		509 (40)				11,135 (43.2)		N/A			N/A	
	Female	6752 (61.5)		8633 (53.8)		1.35 (1.27-1.43)			<.001		764 (60)				14,621 (56.8)		1.16 (1.01-1.33)			.03	
Age (years), mean (SD)			58.5 (11.7)		58.8 (12.3)		1.00 (1.00-1.01)			.03		58.6 (11.3)				58.6 (12.1)		1.00 (1.00-1.01)			.26
IMD quintile^d^																					
	1 (baseline)	1876 (17.8)		3044 (19.6)		N/A			N/A		201 (16.3)				4719 (19)		N/A			N/A	
	2	2022 (19.2)		3007 (19.4)		1.06 (0.97-1.17)			.19		237 (19.2)				4792 (19.3)		1.05 (0.84-1.31)			.66	
	3	2246 (21.3)		3157 (20.3)		1.15 (1.05-1.26)			.003		253 (20.5)				5150 (20.7)		1.04 (0.84-1.29)			.72	
	4	2232 (21.2)		3369 (21.7)		1.07 (0.98-1.18)			.13		262 (21.3)				5339 (21.5)		1.03 (0.83-1.28)			.77	
	5	2163 (20.5)		2966 (19.1)		1.17 (1.06-1.28)			.001		279 (22.7)				4850 (19.5)		1.19 (0.96-1.47)			.12	
Ethnicity																					
	Asian	456 (5.7)		1379 (10.8)		0.51 (0.46-0.57)			<.001		34 (3.7)				1801 (9.1)		0.37 (0.26-0.53)			<.001	
	Black	236 (3)		626 (4.9)		0.57 (0.49-0.67)			<.001		16 (1.7)				846 (4.3)		0.36 (0.22-0.60)			<.001	
	Mixed	69 (0.9)		159 (1.2)		0.62 (0.46-0.83)			.001		7 (0.8)				221 (1.1)		0.62 (0.29-1.32)			.21	
	Other	65 (0.8)		137 (1.1)		0.77 (0.57-1.04)			.08		4 (0.4)				198 (1)		0.42 (0.16-1.13)			.09	
	White (baseline)	7141 (89.6)		10,487 (82)		N/A			N/A		871 (93.5)				16,757 (84.5)		N/A			N/A	
Years since diagnosis, mean (SD)			5.4 (6.5)		6.9 (7.1)		0.97 (0.97-0.98)			<.001		4.6 (6.4)				6.4 (6.9)		0.96 (0.95-0.97)			<.001
How the user was made aware of Healthy Living																					
	Health professional (baseline)	5107 (45.8)		7751 (47.6)		N/A			N/A		646 (50)				12,212 (46.7)		N/A			N/A	
	Social media	1802 (16.2)		3251 (20)		0.81 (0.75-0.88)			<.001		141 (10.9)				4912 (18.8)		0.52 (0.42-0.65)			<.001	
	Online search	2172 (19.5)		2584 (15.9)		1.16 (1.07-1.26)			<.001		259 (20.1)				4499 (17.2)		1.12 (0.94-1.34)			.20	
	Poster/leaflet	476 (4.3)		905 (5.6)		0.88 (0.77-1.01)			.06		63 (4.9)				1318 (5)		1.00 (0.74-1.35)			.99	
	Other	1590 (14.3)		1782 (11)		1.16 (1.05-1.28)			.002		182 (14.1)				3190 (12.2)		1.05 (0.85-1.30)			.64	

^a^Defined as reaching the end of the first section in the Learn Journey.

^b^Defined as accessing at least 60% of the Learn Journey.

^c^N/A: not applicable.

^d^IMD: Index of Multiple Deprivation. IMD scores associated with the lower super output area derived from venue postcodes, ranging from the most deprived areas in England to the least deprived areas in England.

## Discussion

### Principal Findings

Less than half of Healthy Living users who activated an account spent more than 5 minutes on the program. Furthermore, less than half of Healthy Living users reached the end of the first section of the structured Learn Journey content, and very few users “completed” the program, that is, accessed 60% of the structured Learn Journey content. Of those who accessed some program content, only a third of Healthy Living users accessed the unstructured Find Answers section at least once, and even fewer users accessed the Tools section. Hence, while a small proportion accessed a large amount of content, many visited the program only once. Female gender, lower deprivation, White ethnicity, and a shorter time since diagnosis were associated with increased usage.

### Comparisons With Prior Work

Although the measures of usage are similar but not directly comparable, usage appears to be lower than in the HeLP-Diabetes RCT, where the mean number of log-ins in the intervention group was 18.4 (SD 84.0) over a 12-month follow-up [14], compared to the mean number of sessions completed in Healthy Living being 3.1 (SD 19.9). This difference may reflect the tendency for usage to be higher in RCT settings than in the real-world, due to factors such as participant motivation and additional support provided during trials [21]. In addition, previous research from this program of work identified key changes implemented during the national rollout of Healthy Living that contrasted with the RCT, comprising (1) the inclusion of a structured web-based learning curriculum (the “Learn Journey”) due to changes in the NHS policy, (2) a lack of support provided from health care professionals to access the program due to fewer resources in general practice, and (3) the omission of a moderated online support forum due to low uptake of this feature in the HeLP-Diabetes RCT [17]. These changes made during the national rollout may have also contributed to the reasons for lower engagement with Healthy Living.

The results are more comparable to usage analyses in the observational studies of the HeLP-Diabetes: Starting Out program (a structured version of the original HeLP-Diabetes) [24] and the myDESMOND program [22]. In HeLP Diabetes: Starting Out, 23.8% of those who registered reached the end of the first section of structured content, while this percentage was 40.7% for Healthy Living. However, 9.4% of people who registered for HeLP Diabetes: Starting Out completed the program, compared to a completion rate of 4.7% for Healthy Living, although the former was a considerably shorter program [24]. In myDESMOND, 56.3% of users used it for at least a month, and the median number of log-ins was 8 (IQR 4-18) [22], while the median number of sessions completed by users in Healthy Living was 1 (IQR 1-3).

The myDESMOND analysis also found that usage was higher in those aged over 50 years, compared to those aged under 50 years; those aged over 50 years had a higher number of log-ins and spent more time on the program [22]. Similar to our results, the analysis of the myDESMOND program found that usage was higher in females and participants with White ethnicity, although the measures of usage were different. Specifically, females had a similar number of log-ins to males but spent more time on the program, and people with Black or Asian ethnicity had fewer log-ins and spent less time on the program compared to White people [22]. If these differences in usage continue to exist, digital DSMES services risk worsening inequalities based on ethnicity and socioeconomic status, by excluding those groups of society most in need of self-management support. Tailoring to cultural or other specific needs can improve the acceptability and effectiveness of DSMES programs in underrepresented groups [10]; a study of 1678 adults who attended the DESMOND face-to-face program in Leicestershire, including tailoring, identified that 28% of attendances were of South Asian ethnicity, comparable to the 28.3% seen in the local population [25].

### Implications

Potential factors contributing to the low usage of Healthy Living could be the structured nature of the content and the lack of interactive elements. NICE guidelines recommend that DSMES programs be structured to ensure participants are exposed to essential components of the program [1]. However, some users have stated they would have preferred to select topics of interest and felt disengaged when a topic was not relevant to them [26]. A way to overcome this could be to allow users the flexibility to access the content they would find most useful, rather than being directed to work through a curriculum. Additionally, the Healthy Living program was self-led and did not involve interaction with health care professionals or peers, unlike HeLP-Diabetes [17], and there is some evidence from similar programs to suggest that usage and outcomes could be improved by brief human interaction [27-30]. Articles with the highest engagement based on time spent on the page were those with a quiz or video, rather than text only. This is in line with findings from interviews with Healthy Living participants, who said they valued interactive components to keep them engaged [26]. The interviews also found that use of the “Tools” section may have been low because users already had other ways of tracking lifestyle factors, such as mobile apps and wearable technology [26].

Our wider program of research has found that the mean reduction in HbA_1c_ was greater in participants with higher engagement in the program (Zghebi et al, unpublished data, 2025). Results from studies of programs aiming to encourage weight loss have also found that higher levels of usage are associated with better health outcomes [31-33]. For example, 1 trial found that participants who demonstrated consistent use of a weight loss maintenance web-based intervention were more successful at maintaining long-term weight loss [32], and another trial found that greater use of a weight tracker tool was associated with greater weight loss [33]. Given that the NHS 10-Year Plan has committed to shifting care from analog to digital and expanding access to digital self-management tools [11], there needs to be more of a focus on bridging the gap between uptake and engagement with digital programs when implementing at scale.

To explore ways to increase usage with digital programs, future research could explore introducing prompts via SMS text message [34], having more emphasis on unstructured components, or providing more interactive features in the content, such as quizzes and videos. Work could also be done aiming to increase usage specifically in males, those with a Black, Asian, or mixed ethnicity, those from more deprived areas, and people who are not newly diagnosed with type 2 diabetes. Our program of research has found that the program is likely to be cost-effective compared to usual care (Paterson et al, unpublished data, 2025) and therefore could be a viable self-management option to support individuals and to reduce NHS costs for type 2 diabetes treatment and related complications.

### Strengths and Limitations

This was an independent evaluation of a nationally implemented digital DSMES program. Data from all users of Healthy Living over a 3-year period were available, and all user activities were recorded, hence we provided a fine-grained and detailed analysis of usage, which is not commonly done. However, the Healthy Living program was in an early stage at the start of the data analysis period, and changes have been made, such as the removal and addition of some sections of information, which may have had an impact on usage. Furthermore, the study period covers the COVID-19 lockdowns, which may have impacted how much people engaged with the program. Two of the variables included in the analyses assessing associations between user characteristics and usage of the program had high levels of missingness: ethnicity and time since diagnosis. We did not perform multiple imputation, despite it being plausible that the data are missing at random or missing not at random, due to the small number of variables. However, a similar analysis as part of our wider body of work using a dataset with more variables (Zghebi et al, unpublished data, 2025) had similar findings.

### Conclusions

This analysis explored the usage of a nationally implemented digital DSMES program for >27,000 users with type 2 diabetes and demonstrates the importance of considering usage alongside uptake and effectiveness in any evaluation. Unlike previous evaluations, which have focused on smaller sample sizes, this study offers large-scale, real-world evidence on how people actually engage with a national digital DSMES program. As higher usage is associated with improved health outcomes, encouraging increased engagement with the program has the potential to lead to better health outcomes in people with type 2 diabetes.

## Appendix

Multimedia Appendix 1 Screenshots of the 3 main components in the Healthy Living website.

Multimedia Appendix 2 Exclusion criteria.

Multimedia Appendix 3 Engagement with Learn Journey sections.

Multimedia Appendix 4 Articles with the highest engagement.

Multimedia Appendix 5 Most commonly accessed articles in the Find Answers section.

## Abbreviations

DSMES: diabetes self-management education and support
HbA1c: hemoglobin A1c
NHS: National Health Service
NICE: National Institute for Health and Care Excellence
RCT: randomized controlled trial

## Data Availability

The datasets generated or analyzed during this study are not publicly available as they were used under license from NHS England for this study only.

## References

[1] (2022). Type 2 diabetes in adults: management: [NG28]. National Institute for Health and Care Excellence.

[2] Powers MA, Bardsley J, Cypress M, Duker P, Funnell MM, Fischl AH, Maryniuk MD, Siminerio L, Vivian E (2016). Diabetes self-management education and support in type 2 diabetes: a joint position statement of the American Diabetes Association, the American Association of Diabetes Educators, and the Academy of Nutrition and Dietetics. Clin Diabetes.

[3] Chatterjee S, Davies MJ, Heller S, Speight J, Snoek FJ, Khunti K (2018). Diabetes structured self-management education programmes: a narrative review and current innovations. Lancet Diabetes Endocrinol.

[4] Powers M, Bardsley J, Cypress M, Funnell MM, Harms D, Hess-Fischl A, Hooks B, Isaacs D, Mandel ED, Maryniuk MD, Norton A, Rinker J, Siminerio LM, Uelmen S (2020). Diabetes Care.

[5] Odgers-Jewell K, Ball LE, Kelly JT, Isenring EA, Reidlinger DP, Thomas R (2017). Effectiveness of group-based self-management education for individuals with type 2 diabetes: a systematic review with meta-analyses and meta-regression. Diabet Med.

[6] (2024). National Diabetes Audit (NDA) 2023-24 quarterly report. National Health Service.

[7] Davies MJ, Heller S, Skinner TC (2008). Effectiveness of the diabetes education and self management for ongoing and newly diagnosed (DESMOND) programme for people with newly diagnosed type 2 diabetes: Cluster randomised controlled trial. BMJ.

[8] Deakin TA, Cade JE, Williams R, Greenwood DC (2006). Structured patient education: the diabetes X-PERT programme makes a difference. Diabet Med.

[9] Horigan G, Davies M, Findlay-White F, Chaney D, Coates V (2017). Reasons why patients referred to diabetes education programmes choose not to attend: a systematic review. Diabet Med.

[10] Hadjiconstantinou M, Quinn LM, Tippins F, Schreder S, Khunti K, Davies MJ (2021). A perspective piece on diabetes self-management education and support (DSMES) programmes for under-represented groups with T2DM in the UK. Br J Diabetes.

[11] Fit for the future: 10 year health plan for England. NHS England.

[12] Nkhoma DE, Soko CJ, Bowrin P, Manga YB, Greenfield D, Househ M, Li Jack Y, Iqbal U (2021). Digital interventions self-management education for type 1 and 2 diabetes: a systematic review and meta-analysis. Comput Methods Programs Biomed.

[13] Dack C, Ross J, Stevenson F, Pal K, Gubert E, Michie S, Yardley L, Barnard M, May C, Farmer A, Wood B, Murray E (2019). A digital self-management intervention for adults with type 2 diabetes: combining theory, data and participatory design to develop HeLP-Diabetes. Internet Interv.

[14] Murray E, Sweeting M, Dack C, Pal K, Modrow K, Hudda M, Li J, Ross J, Alkhaldi G, Barnard M, Farmer A, Michie S, Yardley L, May C, Parrott S, Stevenson F, Knox M, Patterson D (2017). Web-based self-management support for people with type 2 diabetes (HeLP-Diabetes): randomised controlled trial in English primary care. BMJ Open.

[15] Li J, Parrott S, Sweeting M, Farmer A, Ross J, Dack C, Pal K, Yardley L, Barnard M, Hudda M, Alkhaldi G, Murray E (2018). Cost-effectiveness of facilitated access to a self-management website, compared to usual care, for patients with type 2 diabetes (HeLP-Diabetes): randomized controlled trial. J Med Internet Res.

[16] (2019). Healthy living service specification. NHS England.

[17] Benton JS, Cotterill S, Hawkes RE, Miles LM, French DP (2022). Changes in a digital type 2 diabetes self-management intervention during national rollout: mixed methods study of fidelity. J Med Internet Res.

[18] Nahum-Shani I, Yoon C (2024). Towards the science of engagement with digital interventions. Curr Dir Psychol Sci.

[19] Hardman R, Begg S, Spelten E (2020). What impact do chronic disease self-management support interventions have on health inequity gaps related to socioeconomic status: a systematic review. BMC Health Serv Res.

[20] McLeod M, Stanley J, Signal V, Stairmand J, Thompson D, Henderson K, Davies C, Krebs J, Dowell A, Grainger R, Sarfati D (2020). Impact of a comprehensive digital health programme on HbA and weight after 12 months for people with diabetes and prediabetes: a randomised controlled trial. Diabetologia.

[21] Baumel A, Edan S, Kane JM (2019). Is there a trial bias impacting user engagement with unguided e-mental health interventions? A systematic comparison of published reports and real-world usage of the same programs. Transl Behav Med.

[22] Barker MM, Chauhan R, Davies MJ, Brough C, Northern A, Stribling B, Schreder S, Khunti K, Hadjiconstantinou M (2023). User retention and engagement in the digital-based diabetes education and self-management for ongoing and newly diagnosed (myDESMOND) program: descriptive longitudinal study. JMIR Diabetes.

[23] (2019). The English indices of deprivation. Department for Communities and Local Government.

[24] Poduval S, Marston L, Hamilton F, Stevenson F, Murray E (2020). Feasibility, acceptability, and impact of a web-based structured education program for type 2 diabetes: real-world study. JMIR Diabetes.

[25] Chatterjee S, Davies Mj, Stribling B, Farooqi A, Khunti K (2018). Real‐world evaluation of the DESMOND type 2 diabetes education and self‐management programme. Practical Diabetes.

[26] Hawkes RE, Benton JS, Cotterill S, Sanders C, French DP (2024). Service users' experiences of a nationwide digital type 2 diabetes self-management intervention (Healthy Living): qualitative interview study. JMIR Diabetes.

[27] Hawkes RE, Miles LM, Ainsworth B, Ross J, Meacock R, French DP (2023). Engagement with a nationally-implemented digital behaviour change intervention: usage patterns over the 9-month duration of the National Health Service Digital Diabetes Prevention Programme. Internet Interv.

[28] Ross J, Hawkes RE, Miles LM, Cotterill S, Bower P, Murray E (2023). Design and early use of the nationally implemented Healthier You National Health Service Digital Diabetes Prevention programme: mixed methods study. J Med Internet Res.

[29] Ross J, Cotterill S, Bower P, Murray E (2023). Influences on patient uptake of and engagement with the National Health Service Digital Diabetes Prevention Programme: qualitative interview study. J Med Internet Res.

[30] Dennison L, Morrison L, Lloyd S, Phillips D, Stuart B, Williams S, Bradbury K, Roderick P, Murray E, Michie S, Little P, Yardley L (2014). Does brief telephone support improve engagement with a web-based weight management intervention? Randomized controlled trial. J Med Internet Res.

[31] Cussler EC, Teixeira PJ, Going SB, Houtkooper LB, Metcalfe LL, Blew RM, Ricketts JR, Lohman J, Stanford VA, Lohman TG (2008). Maintenance of weight loss in overweight middle-aged women through the internet. Obesity (Silver Spring).

[32] Funk KL, Stevens VJ, Appel LJ, Bauck A, Brantley PJ, Champagne CM, Coughlin J, Dalcin AT, Harvey-Berino J, Hollis JF, Jerome GJ, Kennedy BM, Lien LF, Myers VH, Samuel-Hodge C, Svetkey LP, Vollmer WM (2010). Associations of internet website use with weight change in a long-term weight loss maintenance program. J Med Internet Res.

[33] Brindal E, Freyne J, Saunders I, Berkovsky S, Smith G, Noakes M (2012). Features predicting weight loss in overweight or obese participants in a web-based intervention: randomized trial. J Med Internet Res.

[34] Bartlett YK, Farmer A, Newhouse N, Miles L, Kenning C, French DP (2022). Effects of using a text message intervention on psychological constructs and the association between changes to psychological constructs and medication adherence in people with type 2 diabetes: results from a randomized controlled feasibility study. JMIR Form Res.

