# Supplementary material for: Usage and Exposure to Content of the NHS Healthy Living Program for People With Type 2 Diabetes: Retrospective Observational Cohort Study
Source: J Med Internet Res. 2026 Jun 2;28:e89690. doi: 10.2196/89690 (PMC13273227; doi:10.2196/89690)

**Multimedia Appendix 1: Screenshots of the three main components in the Healthy Living website: ‘Learn Journey’ (top), ‘Find Answers’ (middle) and ‘Tools’ (bottom).**

Note: These screenshots are for illustration purposes only. They demonstrate how the website looked at the time the research evaluation started in 2020; changes have since been made to the website.


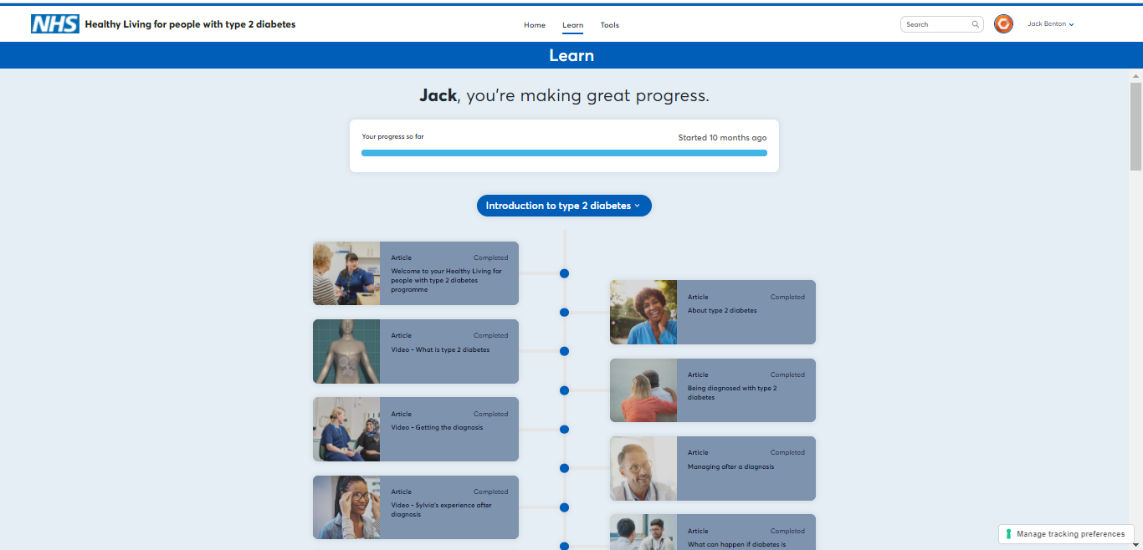


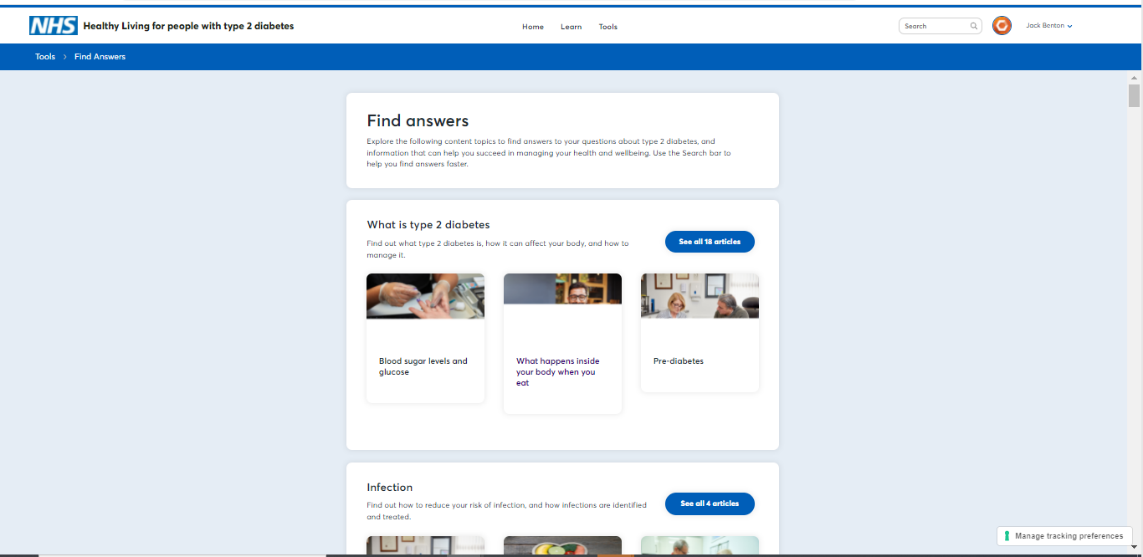


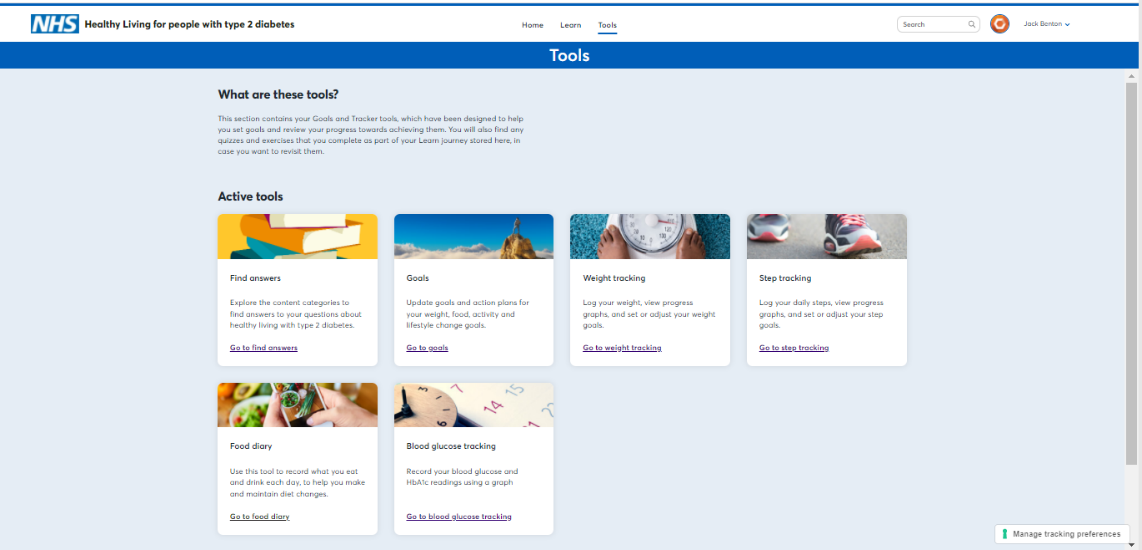

Supplement: Multimedia Appendix 1 [file jmir_v28i1e89690_app1.docx]
