# Supplementary material for: Usage and Exposure to Content of the NHS Healthy Living Program for People With Type 2 Diabetes: Retrospective Observational Cohort Study
Source: J Med Internet Res. 2026 Jun 2;28:e89690. doi: 10.2196/89690 (PMC13273227; doi:10.2196/89690)
Supplement: Multimedia Appendix 2 [file jmir_v28i1e89690_app2.docx]

**Multimedia Appendix 2: Exclusion criteria**

This sensitivity analysis explores the suitability of the exclusion criterion whereby users who activated their account less than one month before data extraction were excluded. The main summaries are repeated, this time excluding users who activated their account 1) less than 3 months before data extraction, 2) less than 6 months before data extraction and 3) less than 9 months before data extraction.

The summaries are similar across all scenarios suggesting the choice of 1, 3, 6 or 9 months does not impact the overall findings.

*How many sessions did participants complete?*

**Table S1 Summary of the number of sessions per users under different exclusion criteria regarding date of account activation**

|  |  |  |
| --- | --- | --- |
| **Excluding participants with less than 3 months of data** | **N**  Mean (SD)  Median (IQR)  Range  1 session completed  2-4 sessions completed  5-9 sessions completed  10+ sessions completed | 24157  3.1 (20.7)  1 (1-3)  1 – 1929  13307 (55.1%)  8048 (33.3%)  1907 (7.9%)  895 (3.7%) |
| **Excluding participants with less than 6 months of data** | **N**  Mean (SD)  Median (IQR)  Range  1 session completed  2-4 sessions completed  5-9 sessions completed  10+ sessions completed | 20212  3.1 (22.6)  1 (1-3)  1 – 1929  11244 (55.6%)  6670 (33.0%)  1558 (7.7%)  740 (3.7%) |
| **Excluding participants with less than 9 months of data** | **N**  Mean (SD)  Median (IQR)  Range  1 session completed  2-4 sessions completed  5-9 sessions completed  10+ sessions completed | 12511  3.5 (26.8)  1 (1-3)  1 – 1929  6779 (54.2%)  4127 (33.0%)  1067 (8.5%)  538 (4.3%) |

*In total, how much time did participants spend on the website?*

**Table S2 Summary of the total time spent on the website (in minutes) under different exclusion criteria regarding date of account activation**

|  |  |  |
| --- | --- | --- |
| **Excluding participants with less than 3 months of data** | **N**  Mean (SD)  Median (IQR)  Range  <1 minute  1-5 minutes  5-30 minutes  30-60 minutes  60-120 minutes  120 minutes + | 24157  24.7 (60.0)  7.4 (0.5-27.6)  0 – 3410.9  6828 (28.3%)  3941 (16.3%)  7747 (32.1%)  2951 (12.2%)  1785 (7.4%)  905 (3.8%) |
| **Excluding participants with less than 6 months of data** | **N**  Mean (SD)  Median (IQR)  Range  <1 minute  1-5 minutes  5-30 minutes  30-60 minutes  60-120 minutes  120 minutes + | 20212  24.6 (62.7)  6.6 (0.3-27.0)  0 – 3410.9  6001 (29.7%)  3351 (16.6%)  6219 (30.8%)  2415 (12.0%)  1469 (7.3%)  757 (3.8%) |
| **Excluding participants with less than 9 months of data** | **N**  Mean (SD)  Median (IQR)  Range  <1 minute  1-5 minutes  5-30 minutes  30-60 minutes  60-120 minutes  120 minutes + | 12511  27.3 (70.5)   - 1. (0.2-30.3)   0– 3410.9  3664 (29.3%)  1951 (15.6%)  3742 (29.9%)  1597 (12.8%)  999 (8.0%)  558 (4.5%) |

*How many participants accessed content and reached milestones?*

**Table S3 Summary of number of participants who accessed content, ‘attended’ and ‘completed’ the programme under different exclusion criteria regarding date of account activation**

| **Excluding participants with less than 3 months of data** | **N**  Accessed structured content  Accessed educational content  Accessed any content  ‘Attended’ the programme  ‘Completed’ the programme | 24157  15305 (63.4%)  16295 (67.5%)  16588 (68.7%)  9535 (39.5%)  1167 (4.8%) |
| --- | --- | --- |
| **Excluding participants with less than 6 months of data** | **N**  Accessed structured content  Accessed educational content  Accessed any content  ‘Attended’ the programme  ‘Completed’ the programme | 20212  12375 (61.2%)  13189 (65.3%)  13445 (66.5%)  7395 (36.6%)  979 (4.8%) |
| **Excluding participants with less than 9 months of data** | **N**  Accessed structured content  Accessed educational content  Accessed any content  ‘Attended’ the programme  ‘Completed’ the programme | 12511  7693 (61.5%)  8159 (65.2%)  8346 (66.7%)  4350 (34.8%)  659 (5.3%) |
