# Supplementary material for: Usage and Exposure to Content of the NHS Healthy Living Program for People With Type 2 Diabetes: Retrospective Observational Cohort Study
Source: J Med Internet Res. 2026 Jun 2;28:e89690. doi: 10.2196/89690 (PMC13273227; doi:10.2196/89690)
Supplement: Multimedia Appendix 3 [file jmir_v28i1e89690_app3.docx]

**Multimedia Appendix 3: Engagement with Learn Journey sections**

Table S4 lists the sections of the Learn Journey and gives the number and percentage of users who accessed that section.

**Table S4 Summary of the number of people who accessed each section of the Learn Journey**

|  | **N(%)** |
| --- | --- |
| 01 - Introduction to type 2 diabetes | 17558 (91.8%) |
| 02 - Complete your profile | 10910 (57.0%) |
| 03 - Self assessment | 9558 (50.0%) |
| 04 - Eating well for diabetes | 7383 (38.6%) |
| 05 - Becoming more active | 4297 (22.5%) |
| 06 - Taking control (Non-smoker) | 2793 (14.6%) |
| 07 - Taking control (Smoking) | 344 (1.80%) |
| 08 - Protecting my body and mind | 2569 (13.4%) |
| 09 - Handling feelings | 2190 (11.4%) |
| 10 - Making changes | 1998 (10.4%) |
| 11 - Making the most of the NHS | 1882 (9.8%) |
| 12 – Medication | 1641 (8.6%) |
| 13 - Reducing risk of heart attacks and strokes | 1612 (8.4%) |
| 14 - Your goals and plans | 1482 (7.7%) |
| 15 - Understanding my moods | 1468 (7.7%) |
| 16 - My diabetes review | 114 (0.6%) |
| 17 - Looking after my feet | 1285 (6.7%) |
| 18 - Review my goals and plans | 1245 (6.5%) |
| 19 - Self assessment | 1155 (6.0%) |
| 20 - Staying motivated | 1192 (6.2%) |
| 21 - Working with health professionals | 1145 (6.0%) |
| 22 - Managing diabetes when you are ill (employed, driver) | 327 (1.7%) |
| 23 - Managing diabetes when you are ill (employed, non-driver) | 75 (0.4%) |
| 22 - Managing diabetes when you are ill (unemployed, driver) | 317 (1.7%) |
| 23 - Managing diabetes when you are ill (unemployed, non-driver) | 388 (2.0%) |
| 26 - Diabetes and my social life (employed, driver) | 294 (1.5%) |
| 27 - Diabetes and my social life (employed, non-driver) | 68 (0.4%) |
| 28 - Diabetes and my social life (unemployed, driver) | 290 (1.5%) |
| 29 - Diabetes and my social life (unemployed, non-driver) | 332 (1.7%) |
| 30 - Working with diabetes | 366 (1.9%) |
| 31 - Driving with diabetes | 948 (5.0%) |
| 32 - Review my goals and plans | 936 (4.9%) |
| 33 - Managing my moods^a^ | 124 (1.7%) |
| 34 - Living life to the full (article) | 902 (4.7%) |

^a^ This section was removed as the content was deemed to be a limited use.
