# Supplementary material for: Usage and Exposure to Content of the NHS Healthy Living Program for People With Type 2 Diabetes: Retrospective Observational Cohort Study
Source: J Med Internet Res. 2026 Jun 2;28:e89690. doi: 10.2196/89690 (PMC13273227; doi:10.2196/89690)
Supplement: Multimedia Appendix 4 [file jmir_v28i1e89690_app4.docx]

**Multimedia Appendix 4: Articles with the highest engagement**

The following articles had a median duration of 90 seconds or higher:

- Section 1, Understanding type 2 diabetes (Quiz)
  Median (IQR): 106 (34, 172)
- Section 3, Find out how confident you feel about managing your diabetes? (Quiz)
  Median (IQR): 121 (28, 189)
- Section 4, A Healthy diet (Video)
  Median (IQR): 129 (32, 229)
- Section 4, Calories and weight (Quiz)
  Median (IQR): 90 (40, 135)
- Section 4, Carbohydrates and blood glucose (Quiz)
  Median (IQR): 92 (20, 165)
- Section 15, Find out how you have been feeling (Quiz)
  Median (IQR): 105 (69, 146)
- Section 19, Find out how confident you feel about managing your diabetes (Quiz)
  Median (IQR): 95 (32, 143)
