# Supplementary material for: Usage and Exposure to Content of the NHS Healthy Living Program for People With Type 2 Diabetes: Retrospective Observational Cohort Study
Source: J Med Internet Res. 2026 Jun 2;28:e89690. doi: 10.2196/89690 (PMC13273227; doi:10.2196/89690)
Supplement: Multimedia Appendix 5 [file jmir_v28i1e89690_app5.docx]

**Multimedia Appendix 5: Most commonly accessed articles in the Find Answers section**

**Table S5 Find Answers articles accessed by more than 600 participants**

| **Article title** | **Section** | **N(%)** |
| --- | --- | --- |
| A healthy balanced diet | Eating well for type 2 diabetes | 799 (4.18%) |
| Calories | Eating well for type 2 diabetes | 683 (3.57%) |
| Carbohydrates | Eating well for type 2 diabetes | 797 (4.16%) |
| Fatty and sugary foods and drinks | Eating well for type 2 diabetes | 701 (3.66%) |
| Foods specifically for people with diabetes | Eating well for type 2 diabetes | 653 (3.41%) |
| Foods to eat and avoid | Eating well for type 2 diabetes | 1091 (5.70%) |
| Fruit and vegetables | Eating well for type 2 diabetes | 839 (4.38%) |
| Healthy portion plate | Eating well for type 2 diabetes | 844 (4.41%) |
| How the body uses food and drink | Eating well for type 2 diabetes | 741 (3.87%) |
| Lower carbohydrate plate | Eating well for type 2 diabetes | 832 (4.35%) |
| Meals and portion sizes | Eating well for type 2 diabetes | 1148 (6.00%) |
| Milk and dairy products | Eating well for type 2 diabetes | 718 (3.75%) |
| Non-dairy sources of protein | Eating well for type 2 diabetes | 745 (3.89%) |
| Snacks and desserts | Eating well for type 2 diabetes | 636 (3.32%) |
| Why we need to eat and drink | Eating well for type 2 diabetes | 789 (4.12%) |
